# Single-Cell Transcriptional Analysis Deciphers the Inflammatory Response of Skin-Resident Stromal Cells

**DOI:** 10.3389/fsurg.2022.935107

**Published:** 2022-06-14

**Authors:** Baoyi Liu, Ang Li, Jingkai Xu, Yong Cui

**Affiliations:** ^1^ Department of Dermatology, China–Japan Friendship Hospital, Beijing, China; ^2^ Graduate School of Peking Union Medical College, Chinese Academy of Medical Sciences and Peking Union Medical College, Beijing, China

**Keywords:** scRNA-seq, skin resident cells, skin stromal cells, immunoregulatory axis, cellular communication

## Abstract

The skin is the outermost barrier of the body. It has developed a sophisticated system against the ever-changing environment. The application of single-cell technologies has revolutionized dermatology research and unraveled the changes and interactions across skin resident cells in the healthy and inflamed skin. Single-cell technologies have revealed the critical roles of stromal cells in an inflammatory response and explained a series of plausible previous findings concerning skin immunity. Here, we summarized the functional diversity of skin stromal cells defined by single-cell analyses and how these cells orchestrated events leading to inflammatory diseases, including atopic dermatitis, psoriasis, vitiligo, and systemic lupus erythematosus.

## Introduction

The skin is the body’s outermost barrier and is composed of the epidermis and dermis. It is defined by a multilayered architecture based on diverse cell populations of keratinocytes, melanocytes, fibroblasts, and endothelial cells, as well as various immune cells. Facing the ever-changing environment, it has developed a complex system that responds to stimuli such as pathogen infection, UV exposure, and toxic compounds (1). In this system, skin stromal cells have long been considered passive participants and bystanders in immunoregulation. However, recent studies have revealed that they actively participate in skin inflammatory responses.

RNA sequencing (RNA-seq) technology has long been used to dissect the molecular differences or commonalities of different inflammatory skin diseases (2). Previous bulk RNA sequencing and microarray studies on skin inflammatory diseases have revealed dysregulation of inflammatory and barrier genes, but many cell-specific transcripts often remain masked in whole-skin transcriptomic analyses. In addition, it has been difficult to systematically understand the sophisticated interplay among cells in the skin (3). Sing-cell RNA-seq (scRNA-seq) can capture tissue transcripts at the single-cell resolution and effectively solve this problem (3). The experimental procedure of scRNA-seq mainly includes four steps: sample preparation, cell enrichment, library preparation, and data analysis (4). It has been leveraged in various fields, contributing to novel discoveries and improving our understanding of embryonic medicine, developmental biology, tumor ecosystems, immunology, and inflammatory diseases (5). scRNA-seq studies have elucidated how skin resident cells are involved in skin homeostasis regulation and inflammatory responses and revealed their immunoregulatory role (6–8). However, scRNA-seq transcriptome analysis remains challenging to define cell subpopulations due to the heterogeneity of skin cells during inflammation.

In this review, we recapitulated the main cell populations and their subpopulations of skin-resident stromal cells observed by scRNA-seq technologies in healthy and inflamed human skin and discussed their pathological alterations and “feedforward” role in recruiting immune cells in skin inflammatory diseases, including atopic dermatitis (AD), psoriasis, vitiligo, and systemic lupus erythematosus (SLE). We attempted to highlight current disagreements and consensus on skin stromal cell subsets and functions in inflammatory diseases.

### Skin Resident Cells Identified by scRNA-seq

Defining subsets of cells is an important step in scRNA-seq bioinformatics analysis. The type of obtained cells varied with different collection methods. Full-thickness skin samples contained complete skin cells, while suction blisters would lose information on fibroblasts, endothelial cells, and immune cells in the dermis (9). However, suction blisters were more accessible and often used in the study of AD (7, 10, 11). Most studies on the skin referred to the Human Cell Atlas (12) for cell identification, which could accurately distinguish skin stromal cells. However, when it comes to specific cell subtypes, especially fibroblasts with obvious heterogeneity, it was difficult for researchers to reach a consensus on grouping and naming. We would describe the disagreements and consensus on each cell subset classification in the following. The biological markers used in scRNA-seq studies are listed in Table 1.

**Table 1:** Cell defined marker of skin resident cell in scRNA-seq.

Cell types/subtypes	Marker	Reference
Keratinocytes	KRT1, KRT2, KRT5	6–8, 13–18
Basal keratinocytes	KRT5, KRT14, TP63, ITGA6, COL17A1	6–8, 13–17
Spinous keratinocytes	KRT1, KRT10, DSG1, DSP	6–8, 13–17
Granular keratinocytes	LOR, FLG, SPINK5, KRT2, KLK11	6–8, 13–17
Mitotic keratinocytes	CDK1, PCNA, KI67, UBE2C, TOP2A, TK1, MTK1, TOP2A	10, 17, 18
Channel keratinocytes	GJB2, GJB6, ATP1B3, ATP1A1, ATP1B1, ATP5B, FXYD3	10, 17, 18
Follicular keratinocytes	APOC1, ACSL5, ABCC3, MGST1 APOE, CD200, SOX9, KRT19, KRT6B, KRT17, S100A2	10, 17, 18
Melanocytes	PMEL, TYR, TYRP1, DCT, KIT, MLANA	6, 7, 11
Fibroblasts	COL1A1/A2, DCN, LUM, LUM, DCN, VIM, PDGFRA	13, 14
Papillary fibroblasts	DPP4, FAP, CD90, COL6A5, CD39, APCDD1, HSPB3, WIF1	16, 19–21
Reticular fibroblasts	Dlk1, CD36	19–21
Secretory-reticular fibroblasts	WISP2, SLPI, CTHRC1, MFAP5, TSPAN8	22
Secretory-papillary fibroblasts	APCDD1, ID1, WIF1, COL18A1, PTGDS	22
Mesenchymal fibroblasts	ASPN, POSTN, GPC3, TNN, SFRP1	22
Pro-inflammatory fibroblasts	CCL19, APOE, CXCL2, CXCL3, EFEMP1	22
Type A	ELN, MMP2, QPCT, SFRP2	14
Type B	APOE, C7, CYGB, IGFBP7B	14
Type C	DKK3, TNMD, TNN, SFRP1	14
Vascular endothelial cells	PECAM1, EMCN, SELE, CD93, CLDN5, VWF, CDH5	6, 8, 18
Lymphatic endothelial cells	LYVE1, PDPN, PROX1, CLDN5	6, 8, 18

### Keratinocytes Actively Contributed to Immunopathology in Inflamed Skin

Keratinocytes are the major cells in the epidermis, accounting for 66% of the epidermic cells from suction blisters and 37.96% of the full-thickness skin from skin biopsies in scRNA-seq (13). Keratinocytes were distinguished from other skin cells by cluster-specific expression of keratin1 (KRT1), KRT2, and KRT5 (6–8, 13–18).

Cell subtype identification of keratinocytes was consistent across various papers (6, 7, 13). Three subpopulations were separated corresponding to their stereotyped differentiation process in the normal epidermis: undifferentiated basal keratinocytes had high expression of basal epidermal proteins (KRT5 and KRT14), hemidesmosome molecules [integrin alpha-6, ITGA6; and collagen alpha-1 (XVII) chain, COL17A1] and stemness factor (tumor protein 63, TP63); upper spinous keratinocytes expressed suprabasal cell transcripts (KRT1 and KRT10); and granular keratinocytes expressed KRT2, KLK11, and late differentiation markers (Loricrin, LOR; Filaggrin, FLG; and serine protease inhibitor Kazal-type 5, SPINK5) (6–8, 13–18).

In addition, three subpopulations were unable to be distinguished by the classical differentiation model. The first undergoing proliferation was deemed as mitotic keratinocytes, as characterized by DNA synthesis and cell division transcripts, including cyclin-dependent kinase 1 (CDK1), proliferating cell nuclear antigen (PCNA), KI67, ubiquitin-conjugating enzyme E2 C (UBE2C), DNA topoisomerase 2-alpha (TOP2A), thymidine kinase (TK1), and mitogen-activated protein kinase kinase kinase 4 (MAP3K4). The second was termed “channel” keratinocytes by coordinate elevation of ion channel and cell-cell communication transcripts [gap junction beta (GJB2/6), potassium-transporting ATPase (ATP1B3/1A1/1B1/5B), and FXYD domain-containing ion transport regulator 3 (FXYD3)]. Finally, the third was separated individually as follicular keratinocytes that expressed genes associated with the sebaceous gland [apolipoprotein (APOC1/E), long-chain-fatty-acid–CoA ligase 5 (ACSL5), ATP-binding cassette sub-family C member 3 (ABCC3), and microsomal glutathione S-transferase 1 (MGST1)], follicles (TRK19, CD200, SOX9), and (or) outer root sheath (KRT6B, KRT17, and S100A2) (10, 17, 18).

Interestingly, force-directed graph (FDG) and PAGA analyses on scRNA-seq data defined two differentiation pathways of basal keratinocytes to suprabasal cells, with or without a high level of the lamellar body (LB)-related transcripts (glucosylceramide transporter ABCA12; cytoskeleton-associated protein 4, CKAP4; and CAP-Gly domain-containing linker protein 1, CLIP1), that characterized late epidermal differentiation in healthy skin (6). When it comes to inflammation, keratinocytes showed a more complicated picture of gene expression. Gene differential expression analysis revealed that keratinocytes, especially those in spinous and granular layers, overexpressed a panel of interferon response genes [interferon alpha-inducible protein 27 (IFI27) and interferon-induced transmembrane protein 1 (IFITM1)] and damage-associated transcripts, including antimicrobial peptides, also known as alarmis (S100A7/A8/A9), wound healing states (KRT6A/16), and serpins (SERPINB4/13), consistent with previous study results (4, 6, 13). It was reported that evoked S100A8/A9 expression was a molecular event preceding any histological alteration or deregulation of cytokines and played a crucial role in enhancing keratinocyte differentiation while inhibiting proliferation (23). In addition to damage-associated molecules, keratinocytes also expressed chemokine ligands and cytokines to recruit immune cells. Reynolds et al. defined a new subpopulation of celled inflammatory differentiated keratinocytes, as suggested by coexpressing lower levels of undifferentiated and differentiated markers but additionally expressing intercellular adhesion molecule1 (ICAM1), tumor necrosis factor (TNF), and CCL20 (6). CCL20 is the only known high-affinity ligand of CCR6 and attracts IL17A-producing CCR6+ immune cells, such as dendritic cells (DCs) and T cells (24). It has been reported that the CCL20/CCR6 axis contributes to the formation of an IL17A-rich milieu in psoriasis (25). Pruritus-induced mechanical scratching also upregulated CCL20 production from keratinocytes, which could partially explain high IL17A levels in AD (24).

Several disease-specific alterations were described in keratinocytes of AD patients. Abnormally elevated genes included aquaporin-3 (AQP3, a water channel component associated with keratinocyte proliferation and transepidermal water loss) (26), tumor necrosis factor receptor superfamily member 12A (TNFRSF12A), cornifin-B (SPRR1B, promoting the cornification of keratinocytes) (10, 27), CCL2 (binding to CCR2 to polarize Th2 response) (28), CCL27, and proinflammatory cytokine IL32 (modulating keratinocyte apoptosis) (17, 29). Downregulated genes involved DEFB1, an antimicrobial against *Staphylococcus aureus* (10, 30). After dupilumab treatment, most of the activated transcripts were normalized, including S100A7/A8/A9, KRT6A, KRT16, SERPINB4, CCL2, and CCL27, whereas IFI27, TNFRSF12A, and SPRR1B remained dysregulated 1 year later (31). The relationship between these genes and recurrence is worth exploring.

Psoriatic epidermis is remarkable for the expansion of mitotic and channel subfractions with increased GJB6, which is described as a psoriasis risk gene (18). In addition, keratinocytes significantly overexpress MHC molecules (HLA-DRA, HLA-DMA, HLA-C), WNT5A, CD58, and pro-inflammatory cytokines IL36G and NFKBIZ (transcriptional regulator of IL36-driven gene expression) (13, 32). It has been described that keratinocytes are capable of directly presenting antigen to T cells and initiating an immune response (33). IL36γ could induce keratinocytes to produce CXCL1, CXCL10, and CCL20 and function as a linker between keratinocytes and DCs (34). Unexpectedly, Hughes et al. found that FOS-related antigen 1 (FOSL1) was positively correlated with diffusion pseudo-time in psoriatic KCs compared with healthy KCs by differential pseudo-time correlation analysis and further validated the distribution at the protein level by immunofluorescence staining (8). FOSL1 belongs to the FOS gene family that has been implicated as regulators of cell differentiation, proliferation, and transformation. FOSL1 was identified as the TF encoding gene of psoriasis (35), and the enhanced FOSL1 expression was significantly correlated with high psoriasis area and severity index (36). FOSL1 knockdown inhibited IL22-induced proliferation and enhanced apoptosis of keratinocytes (37). These results suggest that FOSL1 may serve as a new therapeutic target for psoriasis.

It is an intriguing finding that CCL27 expression of keratinocytes was increased in AD (17) but decreased in psoriasis (32). CCL27 is a chemotactic factor binding to CCR10 that plays an important role in the homeostatic establishment of resident lymphocytes (38). CCL27/CCR10-derived regulation would promote activated T cell product IL17A/IL22 (38). Interactions between CCL2 and CCL27 (keratinocytes) and CCR1/2 and CCR10 (T cells) might cooperatively induce the Th2 milieu in AD (17). Nevertheless, it was confusing that impaired CCL27–CCR10 interaction was present in IL17-driven psoriasis (32). This contradictory result suggested that CCL27 might serve as a molecular marker for differentiating psoriasis and AD.

Keratinocytes respond to inflammation even without visible skin damage. It was reported that keratinocytes upregulated IFN-responsive genes and fibrotic pathways in non-lesional, non-sun-exposed skin of SLE (39, 40). Keratinocytes can even recruit CXCR3+ T cells to kill surrounding melanocytes by expressing high levels of chemokines CXCL9/10/11 (16). These results emphasized that keratinocytes collaborate with immune cells and actively contribute to immunopathology.

### Melanocytes Not Bystanders of Skin Inflammation

Melanocytes account for 10% of the basal cells of the epidermis, and they can be captured at a relatively large proportion by suction blistering, defined by PMEL, tyrosinase (TYR), tyrosinase-related protein 1/2 (TYRP1/2), KIT, and melanoma antigen (MLANA) in scRNA-seq. Melanocytes express many molecules necessary for the maintenance of the extracellular matrix and cell–cell adhesion, including fibronectins, collagens, and laminins, and adhesion to the basal keratinocyte layer by expressed cognate ligands (FN1, LAMBA4, and LAMB1) (6, 7, 11).

Pigmentation changes are very common in inflammatory skin diseases. Consistently, scRNA-seq studies indicated that melanocytes display considerable numbers of dysregulated genes, including S100 transcripts and pigmentation-associated genes (*TYR* and *MFSD12*) in inflamed skin. Despite clinical clearance of the skin, these genes are still overexpressed in spontaneously healed or dupilumab-treated AD (7, 11).

Melanocytes were previously seen as bystanders of skin inflammation, but scRNA-seq demonstrated that they are involved in a variety of inflammatory responses. They increase MHC-I signaling transcript (HLA-A, HLA-B, and HLA-E) in inflamed skin, targeting T cells (HLA-CD3D) and natural killer (NK) cells (HLA-E-KLRC1) (16). It was reported that HLA-C-presented melanocyte was attacked by CD8± T cells and further induced T cells to secrete IL17A in psoriasis lesions (41). Notably, HLA-C might also play a crucial role in transformation from acute inflammation to a chronic disease (42). Furthermore, some melanocytes significantly evoked IL4Rα, CCL18, and CXCL1 expression responses to type 2 inflammation in AD (7, 43). In vitiligo, a disease characterized by melanocyte loss, CCL18 was also overexpressed in melanocytes, which attracted CCR8+ immune cells (T and NK cells) to kill themselves (16, 44). These pieces of evidence imply that melanocytes are part of a “feedforward” inflammatory response in the epidermis, resembling keratinocytes.

Despite their pro-inflammatory role, melanocytes might be involved in inflammatory diminishing, as suggested by overexpressing matricellular protein CCN family member 3 (CCN3, limiting proinflammatory activation) (45), SERPINF1 (antiangiogenic factor) (46), and Annexin A1 (ANXA1, an anti-inflammatory factor functioning as a major mediator of glucocorticoid responses) in AD (7, 47). Consistent with this study, Rindler et al. found that melanocytes upregulated T cell reactivity inhibitor dickkopf-related protein 3 (DKK3) (48), TGF-β signaling promotor CD81 (49), and platelet-activating factor acetylhydrolase (PLA2G7), which catalyzed the degradation of the strongly pro-inflammatory phospholipid mediator platelet-activating factor (PAF) (11, 50) in spontaneously healed AD (11). Similarly, anti-inflammatory genes (CCN3 and ANXA1) remained among the top upregulated genes after 1-year dupilumab treatment, whereas inflammatory factor expression was normalized (CCL18 and CXCL1) (31).

### Heterogeneity, Dysregulated Extracellular Matrix, and Immune Regulation of Fibroblasts in Diseases

Fibroblasts make up the majority of dermal cells, marked by collagen alpha-1 (I) chain (COL1A1/A2), decorin (DCN), lumican (LUM), vimentin (VIM), and platelet-derived growth factor receptor alpha (PDGFRA). Fibroblasts play an important role in epidermal stem and progenitor cell (EpSC) maintenance (51, 52) and immune surveillance of the dermis (53). Fibroblasts account for 31.53% of the full-thickness skin cells from skin biopsies (13, 14) but are lost in suction blisters for scRNA-seq.

Fibroblasts can be spatially divided into upper papillary fibroblasts and lower reticular fibroblasts. Driskell et al. first defined dipeptidyl peptidase 4 (DPP4, also termed CD26) as a papillary fibroblast marker and delta-like non-canonical notch ligand 1 (Dlk1)+ cell as a fibroblast within the reticular layers (19). It is interesting that DPP4± fibroblasts deposit the matrix during wound healing in mice (54). In line with this study, Guerrero-Juarez. et al. identified CRABP1± papillary fibroblasts in large wounds by conducting scRNA-seq (55). These studies implied that upper papillary dermal compartments seem to be more actively involved in wound regeneration. Subsequently, fibroblast activation protein (FAP), CD90, α5 chain of collagen VI (COL6A5), CD39, APCDD1, heat shock protein beta-3 (HSPB3), and WNT inhibitory factor 1 (WIF1) were identified as papillary fibroblast markers, whereas FAP− CD90+ CD36+ fibroblasts were classified as reticular fibroblasts (20, 21). Philippeos et Al. revealed distinct properties of the upper and lower dermis using RNA-seq on the dermis separated by microdissection (21). The study demonstrated that the papillary dermis exhibited upregulation of the Wnt signaling pathway (WIF1, APCDD1, RSP01, axin-2, AXIN2) and presented an anti-inflammatory phenotype, as suggested by a significant reduction in the upregulation of PDL-1 and CD40 compared with lower fibroblasts in response to IFNγ stimulation. Reticular layers highly expressed several extracellular matrix (ECM) components (ADAM12, ADAMTS9, COL1A2, COL1A1, ELN, and FN1) and inflammatory mediators (CCL19, CCL7, CXCR4, and IL6) (21).

However, the classical markers rarely distinguish the two populations in scRNA-seq studies. Ascensión et al. conducted an integrated analysis of fibroblasts in four scRNA-seq studies and found that papillary and reticular fibroblast categories were composed of a mixture of cell subtypes (14). The classical papillary marker CD26 transcript was present in virtually all cells in the dermis without regional restriction (56). It might result from the functional heterogeneity of fibroblasts (57) or the progressive loss of clear genetic distinctions during the aging process (22). In addition, the heterogeneity of fibroblastic cells also existed across developmental time, between different anatomic skin locations (58). Therefore, fibroblast subsets identified varied among studies. How to unify the nomenclature of fibroblast subtypes is an urgent problem.

A possible strategy is to combine spatial location and function. Solé-Boldo et al. classified fibroblasts into four subpopulations: secretory-reticular fibroblasts [CCN5, antileukoproteinase, SLPI, collagen triple helix repeat-containing protein 1 (CTHRC1), microfibrillar-associated protein 5 (MFAP5), and tetraspanin-8 (TSPAN8)], secretory-papillary fibroblasts [(APCDD1, ID1, WIF1, COL18A1, prostaglandin-H2 d-isomerase (PTGDS)], mesenchymal fibroblasts [asporin (ASPN), periostin (POSTN), glypican-3 (GPC3), tenascin-N (TNN), and secreted frizzled-related protein 1 (SFRP1)], and pro-inflammatory fibroblasts [CCL19, APOE, CXCL2, CXCL3, and EGF-containing fibulin-like extracellular matrix protein 1 (EFEMP1)] that are widely spread within the dermis (22). Another study re-analyzed the four published datasets and classified fibroblasts into three major subsets: type A accounted for about 49.5% of the fibroblasts (defined by ELN, MMP2, QPCT, and SFRP2) and was responsible for dermal cell and ECM homeostasis; type B represented 30.5% of the fibroblasts (defined by APOE, C7, CYGB, and IGFBP7B) and might play a clear role in immune surveillance and inflammation promotion; and type C represented 49.5% of the fibroblasts (defined by DKK3, TNMD, TNN, and SFRP1) and included more specialized subpopulations, such as dermal papilla cells and dermo-hypodermal junction fibroblasts (14). There were still significant differences between the two methods. To clearly describe the expression changes of fibroblasts, it is necessary to identify function-based cellular markers and arrive at a consensus for nomenclature.

Due to the different classification of fibroblast subsets among various studies, fibroblasts will be considered as a group in discussing their changes in inflammation. As expected, fibroblasts significantly increased the expression of MHC-I and MHC-II molecules (HLA-A, HLA-B, HLA-C, and HLA-DRA) in inflamed conditions (13), driving T and NK cell activation *via* targeting CD4, CD8, and CD94 (59, 60). In addition to antigen presentation, Reynolds et al. found that COL1A1+ COL1A2+ COL6A1+ fibroblasts were significantly enriched in AD and psoriatic conditions, which specialized toward ECM remodeling and maintenance, and overexpressed chemokines CXCL12 and CCL19 (6).

Consistent with the abovementioned study, Gao et al. also demonstrated that ECM-related proteins (SDK1, FGF7, COMP, COL5A3, and COL1A1) were elevated in psoriatic fibroblasts, indicating ECM remodeling in the inflamed condition (13). As major contributors to and regulators of inflammation, psoriatic fibroblasts also significantly increased cytokines CCL26, IL6, LIF, IL17B, CCL19, CXCL12, TNFRSF11B, and TNFSF13B (BAFF) (8, 13). CCL26 was a classical type 2 chemokine, while IL6 was considered a hallmark inflammatory cytokine formed by fibroblasts, and the autocrine LIF-LIFR positive feedback loop was critical for maintaining sustained I-6 transcription (61). Increased IL17B was enriched in perivascular fibroblasts (PDGFRβ+), which might be involved in the surface expression of CD80 and CD86 proteins in DCs (13, 62). CCL19 was one of the ligands of CCR7 that was widely expressed by DCs and T cells (63), suggesting that fibroblasts might communicate with these cells frequently in psoriasis. Elevated BAFF expression was strongly correlated with male psoriatic arthritis (PsA) activity and the progression of rheumatoid arthritis (64, 65), implying a consistent response of fibroblasts to inflammation throughout the body. All these results underlined dysregulated ECM and immune regulation of fibroblasts in psoriasis.

Of note, Rojahn et al. reported a new inflammatory fibroblast subpopulation only present in the upper dermis of AD lesions, marked by COL6A5 and COL18A1. These fibroblasts expressed inflammatory cytokines, including CCL2, CCL19, and IL32, similar to inflammatory keratinocytes. The study further confirmed that fibroblasts were juxtaposed to CD3+ T cells, indicating that these inflammatory fibroblasts were engaged in T cell recruitment and organization (10). The special role of this group of cells is worth studying. In addition, like upper keratinocytes and melanocytes, fibroblasts also secret CXCL9/10 in vitiligo. Xu et al. found that IFNγ-responsive fibroblasts were responsible for melanocyte loss via recruiting CD8± T cells through single-cell analysis, along with cell-type-specific genetic knockouts and engraftment experiments (66). These findings suggested that skin stromal cells induced a specific immune milieu *via* secreting the same regulatory factors in an inflammatory response.

### Endothelial Cells in Regulating Leukocyte Adhesion and Migration

There are few endothelial cells in the dermis that are not even detected in studies. Endothelial cells in the healthy adult dermis constitute the vascular endothelium (PECAM1, EMCN, SELE, CD93, CLDN5, VWF, and CDH5) and lymphatic endothelium (LYVE1, PDPN, PROX1, and CLDN5) (6, 8, 18). Reynolds et al. reported that some vascular endothelial cells, which expressed γ-synuclein (SNCG) and high level of ACKR1 (the venular capillary marker), might function as postcapillary venular cells. They found that ACKR1+ endothelial cells and CXCL8 (IL8)+ macrophages interacted with each other by cell communication analysis and further confirmed this finding with immunofluorescence staining *in situ* (6). Besides, ACKR1+ endothelial cells coexpressed inflammatory cytokines, chemokines, and leukocyte adhesion molecules including IL6, IL33, SELE, and ICAM1, implying that they might regulate leukocyte adhesion and migration (6).

## Conclusion

**Figure 1:**
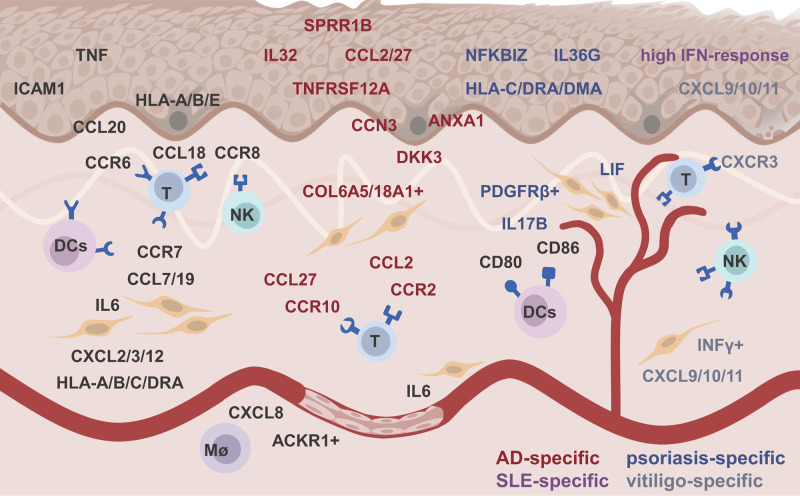
The feedforward role of skin stromal cell in inflamed skin. Black, red, blue, purple, and gray words represented ubiquitous, atopic dermatitis -specific, psoriasis-specific, systemic lupus erythematosus-specific, and vitiligo-specific altered genes, respectively.

Although many technical issues remain to be resolved, scRNA-seq has demonstrated alterations in skin-intrinsic stromal cells in many inflammatory diseases (9). Thanks to scRNA-seq, we can precisely and systematically understand the heterogeneity of skin resident stromal cells, their pathological responses, and their interactions with immune cells in inflamed skin (Table 2). Skin stromal cells played a feedforward role by expressing chemokines, (pro)inflammatory factors, presenting antigens to immune cells, or limiting inflammation by expression regulators (Figure 1). These findings will greatly accelerate the development of personalized diagnostics and precision treatment.

**Table 2:** Molecule changes of skin resident cell in inflamed skin.

Molecular changes	Function	Disease	References
*Keratinocytes*			
IFI27, IFITM1	Interferon response genes	AD and psoriasis	3, 6, 13
S100A7/A8/A9	Early molecular event of inflamed skin	AD and psoriasis	3, 6, 13
KRT6A/16	Wound healing states	AD and psoriasis	3, 6, 13
SERPINB4/13	Protease inhibitor	AD and psoriasis	3, 6, 13
CCL20	Attracting IL17A-producing CCR6 + immune cells	AD and psoriasis	3, 6, 13, 24, 25
AQP3	Involved in keratinocytes proliferation and transepidermal water loss	AD	26
TNFRSF12A	Tumor necrosis factor receptor	AD	10, 27
SPRR1B	Promoting the cornification of keratinocytes	AD	10, 27
CCL2	Binding to CCR2 to polarizing Th_2_ response	AD	28
CCL27	Binding to CCR10 which have important function in homeostatic establishment of resident lymphocytes	AD	17, 38
IL32	Modulating keratinocyte apoptosis	AD	17, 29
DEFB1	An antimicrobial against *Staphylococcus aureus*	AD	10, 30
HLA-DRA, HLA-DMA, HLA-C	Antigen presentation	Psoriasis	13, 32
WNT5A	Regulator of Wnt signaling	Psoriasis	13, 32
CD58	Ligand of the T-lymphocyte CD2 glycoprotein	Psoriasis	13, 32
IL36G	Linker between keratinocytes and DCs	Psoriasis	13, 32, 33
NFKBIZ	Transcriptional regulator of IL36-driven gene expression	Psoriasis	13, 32
FOSL1	Regulators of cell differentiation, proliferation, and transformation	Psoriasis	35–37
CCL27	Binding to CCR10 which have important function in homeostatic establishment of resident lymphocytes	Psoriasis	32, 38
CXCL9/10/11	Recruit CXCR3+ T cells to kill surrounding melanocytes	vitiligo	16
*Melanocytes*			
S100A8/A9	Damage-associated transcripts	AD and psoriasis	7, 11
TYR, MFSD12	Pigmentation-associated genes	AD and psoriasis	7, 11
HLA-A, HLA-B, HLA-E	Antigen presentation	AD and psoriasis	7, 11
IL4Rα, CCL18, CXCL1	Responses to type 2 inflammation	AD	7, 43
CCN3	Limiting proinflammatory activation	AD	7, 45
SERPINF1	Antiangiogenic factor	AD	7, 46
ANXA1	Mediator of glucocorticoid responses	AD	7, 47
DKK3	T-cell reactivity inhibitor	AD	48
PLA2G7	Catalyzing the degradation of the strongly pro-inflammatory phospholipid mediator Platelet-activating factor	AD	11, 50
CCL18	Bind to CCR8+ immune cells (T and NK cell)	Vitiligo	16, 44
*Fibroblast*			
HLA-A, HLA-B, HLA-C, HLA-DRA	Antigen presentation	AD and psoriasis	13
CCL19	Ligands of CCR7 which widely expressed by DCs and T cells	AD and psoriasis	63
CXCL12	CXCR4/CXCL12 axes is involved in asthma pathology (induce a type 2 allergic response)	AD and psoriasis	8, 13, 67
SDK1, FGF7, COMP, COL5A3, COL1A1	ECM-related proteins	Psoriasis	13
CCL26	Classical type 2 chemokines	Psoriasis	61
IL6/LIF	Hallmark inflammatory cytokine made by fibroblasts	Psoriasis	61
IL17B	Involved in surface expression of CD80 and CD86 protein in DCs	Psoriasis	13, 62
CCL2	Binding to CCR2 to Polarizing Th_2_ response	AD	28
IL32	Modulating keratinocyte apoptosis	AD	17, 29
*Vascular endothelial cells*			
ACKR1	Interaction with CXCL8 (IL8)+ macrophages	AD and psoriasis	6

*AD, atopic dermatitis; NK, natural killer cells; ECM, extracellular matrix.*

## Author Contributions

BL drafted and wrote the initial manuscript. AL, JX, and YC reviewed the manuscript. All authors contributed to the article and approved the submitted version.

## Funding

This work was supported by the National Natural Science Foundation of China (Grant No. 81872516, 82173418, and 82103100).

## Conflict of Interest

The reviewer YZ declared a shared parent affiliation with the authors BL, AL, and YC to the handling editor at the time of review.
